# The Effect of Cellulose Nanocrystal Suspension Treatment on Suspension Viscosity and Casted Film Property

**DOI:** 10.3390/polym13132168

**Published:** 2021-06-30

**Authors:** Yucheng Peng, Brian Via

**Affiliations:** School of Forestry and Wildlife Sciences, Auburn University, 520 Devall Drive, Auburn, AL 36849, USA; brianvia@auburn.edu

**Keywords:** cellulose nanocrystal, viscosity, film, homogenization, ultrasonication

## Abstract

Cellulose nanocrystals (CNCs) have attracted significant interest in different industrial sectors. Many applications have been developed and more are being explored. Pre-treatment of the suspension plays a critical role for different applications. In this study, different pre-treatment methods, including homogenization, ultrasonication, and mixing with a magnetic stirrer were applied to a CNC suspension. After treatment, the rheological behaviors of the treated CNC suspensions were characterized using a rotational viscometer. The treated suspensions were then used to cast films for characterization by ultraviolet-visible (UV-Vis) and Fourier transform near-infrared spectroscopy (FT-NIR). All the CNC suspensions demonstrated a shear thinning phenomena. Homogenization or ultrasonication significantly decreased the suspension viscosity compared with the suspension mixed by a magnetic stirrer. The viscosity of CNC suspension changed with time after treatment and settlement of treated CNC suspensions in room conditions increased the viscosity dramatically with time. Different UV and visible light interferences were observed for the CNC films generated from suspensions treated by different methods. The degree of crystallinity of the CNC films evaluated by FT-NIR showed that the film from suspension treated by homogenization and ultrasonication has the highest degree of crystallinity. Pre-treatments of CNC suspension affected the suspension viscosities and formed film properties.

## 1. Introduction

The interest in renewable nanomaterial of cellulose nanocrystals (CNCs) has been growing significantly for the past several decades. Many applications have been developed and more are being explored [1,2,3,4,5,6]. In woody biomass, CNC is the fundamental building block in crystalline structure to form cellulose elementary fibrils (microfibrils/nanofibrils). The cellulose microfibril/nanofibril consists of an additional cellulose amorphous region and the amorphous region arranges alternatively with the crystalline region along the fiber axis [7,8]. Production of CNCs from woody biomass is typically performed through a strong acid hydrolysis of cellulose materials, digesting the amorphous region. In this process, the cellulose source, acid reactant, and hydrolysis parameter impact the surface chemistry and particle morphology of the separated CNC particles. Using sulfuric acid, a stable colloidal suspension of CNCs in water can be prepared and the presence of negatively charged sulfate half-ester groups on the CNC surface prompts a well-dispersion aqueous suspension of CNCs [6,9,10,11].

CNC suspensions demonstrate unique properties due to the dispersion in water of rod or spindle shape particles with the nanometer dimensions in diameter and length [8,12]. At different concentrations, CNC suspensions are known to form either a chiral nematic ordered structure (anisotropic) or a gel-like (random dispersed or isotropic) material [13,14,15,16,17]. The formation of an ordered structure because of self-assembly depends on CNC particle morphology, surface chemistry, and temperature [17,18]. Ionic strength of the suspension can also be changed by adding different ions to control the phase transition between the chiral nematic ordered structure and the gel-like material [19,20]. After drying to form films, the effect of CNC suspension concentration, external shear force, and temperature on properties of casted CNC films were observed to be critical and complex [18,21,22,23]. Simultaneously, the self-assembled structure of CNC particles during the drying process of making film can be fine-tuned and customized to improve film quality, including the properties of optical, physical, mechanical, etc. Intensive research has been conducted in this regard for exploring the formation of multifunctional thin films from CNCs for a variety of applications [24,25,26,27,28,29]. However, the direct correlation between the CNC suspension properties and the formed film properties has not been clearly established. In this study effort was made to have a better understanding of the formation of CNC films through different pre-treatments of a CNC suspension. We treated the CNC suspension first with different methods, changing the suspension properties, mainly on viscosity. The films were then casted from the treated CNC suspensions and were characterized. The effect of CNC suspension viscosity change on the film characteristics was investigated.

Ultrasonication pre-treatment has been commonly employed to treat CNC suspensions for better particles dispersion and distribution [30,31,32,33,34,35,36,37,38,39,40]. The well-recognized functions of ultrasound treatment include better dispersion of individual CNC particles in the suspension [30,33,36], fragmentation of CNC particles in length [31,32,35], and decrease of CNC suspension viscosity [33]. Right after ultrasonication, the film casted from the CNC suspensions has also been investigated. Drying of the CNC suspension with self-assembly structure formed chiral nematic CNC films and the original CNC particle arrangements presented in the suspension maintained in the formed solid film [21,41]. The ultrasonication was observed to impact the CNC particles arrangement in the suspension and during the film drying process. Different ultrasonication treatment parameters shifted the visible light reflection band from the CNC films due to the change of the chiral nematic pitch. A higher ultrasound input energy to the CNC suspension induced a greater chiral nematic pitch for the solid film [42]. The structure change of the ordered chiral nematic domain in the CNC suspension also led to the rheological behavior change of the CNC suspensions [33]. However, the rheological behavior change along time after the ultrasonication treatment has not been recognized and studied. This is a critical factor to be considered when casting films from a CNC suspension if the suspension viscosity keeps changing. The CNC self-assembly nature would theoretically change or recover the effect from ultrasonication treatment. This is especially a concern under the circumstance of reduced CNC suspension viscosity caused by the ultrasonication treatment. The timing casting the CNC film from the suspension after the treatment would also be an important factor to be studied for controlling and optimizing the film quality.

The focus of this research was to characterize the rheological property change of a CNC suspension with time using a rotational viscometer after ultrasonication treatment. The effect of rheological property change of the CNC suspension on the film formation and film properties was also evaluated in this study. Two common mechanical treatment methods in addition to ultrasonication were also included in this study: mechanical stirring using a magnetic stirrer and high shear homogenization. For large-scale industrial applications, processing of suspensions using mechanical method is more widely practiced and can be more conveniently adopted. The rheological information provided in this research would offer significant insights to the industrial partners on processing CNC suspensions for a variety of applications, facilitating product development and market exploration. In this study, we used a rotational viscometer to analyze the effect of different suspension treatments on rheological behaviors of CNC suspensions due to its inexpensive nature and convenient operation in research laboratories and large-scale production plants. The change of viscosity of the CNC suspension was also researched with suspension settlement time. A novel observation was that the CNC suspension viscosity increased with time after each treatment and the viscosity change is closely related to the treatment method and history.

## 2. Materials and Methods

### 2.1. Materials

CNC was purchased in suspension format at 6 wt.% from CelluForce (Montreal, QC, Canada). The suspension was stored in a freezer at 6 °C after receiving.

### 2.2. CNC Suspension Preparation

The CNC suspension was sampled and settled in a sealed high-density polyethylene (HDPE) container at room temperature of 22 ± 1 °C and relative humidity (RH) of 50 ± 5%. After reaching the temperature equilibrium, the suspension was designated as the original CNC suspension (CNC_O). A magnetic stirrer (Corning, Glendale, AZ, USA) was then used to mix the original CNC suspension at room temperature with spin bar rotation at 870 revolution per minute (RPM) for one hour and the CNC suspension obtained was named CNC_M. Ultrasonication treatment of the CNC_M suspension was performed in a sonication bath using a VWR Ultrasonic cleaner (Radnor, PA, USA) for one hour with the container top loosely sealed to minimize the water evaporation from the suspension and the suspension after treatment was designated as CNC_U. Homogenization of the CNC_M suspension was conducted through an IKA disperser T18 digital Ultra-turrax (Wilmington, NC, USA) at 15,000 rpm for 2 min. After homogenization, air bubbles were introduced into the suspension and magnetic stirrer mixing for another hour at 870 RPM was used to eliminate the air bubbles. The obtained suspension was designated as CNC_H. The homogenized CNC suspension was also treated additionally with ultrasonication for another hour to eliminate the air bubbles and the suspension was then named as CNC_H+U. After the treatment, all the suspensions were settled in closed HDPE containers at room conditions for overnight for next step operation.

### 2.3. Rheological Property Characterization

The rheological behaviors of the CNC suspensions were characterized using an IKA Rotavisc lo-vi viscometer (Wilmington, NC, USA). A cylindrical spindle SP1 with a spindle guard leg was used for the apparent viscosity measurement in a 600 mL low form beaker at room temperature 22 ± 1 °C. The spindle SP1 has a dimension of 18.8 mm in diameter and 65.1 mm in length. The CNC suspension around 600 mL was required for each measurement. The suspension holding beaker has an inner diameter of 84.5 mm. The viscometer can rotate the spindle with a continuous speed change from 0 to 200 RPM. The measurement temperatures were recorded by the viscometer and were observed to be changing within 1 °C of the room temperature. During the measurement, spindle rotation speeds were controlled with the torque readings changing from around 10% to 90% of the full-scale torque of this viscometer which is 673.7 dyne-centimeters. The primary readings from this experiment include the torque percentage and the spindle rotation speed in RPM. The torque percentage were recorded when the reading from the viscometer became stabilized and it took less than 1 min to get one data point. Different spindle rotation speeds were generated during the measurement to obtain enough data points for a curve fitting in Excel to characterize the rheological behaviors of the CNC suspensions. Depending on the viscosities of different CNC suspensions, the spindle rotation speeds were in different ranges and the rheological properties characterized will be limited in that shear rate range. The applied torques were in the similar range from around 10% to 90%.

The rheological characterization was performed on all the CNC suspensions settled overnight at room temperature in closed HDPE containers. Prior to the apparent viscosity measurement, the suspension was poured into a 600 mL beaker carefully, avoiding introducing air bubbles into the suspension. After the initial viscosity measurement, the CNC suspensions treated by different methods were then allowed to settle at room temperature for different time periods for follow-up apparent viscosity measurements. The first viscosity measurement after setting the suspension overnight was designated as the data from day one. Then, the suspension viscosity measurement conducted on day five means the suspension has been settled for five days since the treatment. The stability of the CNC suspensions from the perspective of apparent viscosity was investigated.

### 2.4. CNC Film Preparation

The CNC_O suspension was cast directly into a polystyrene Petri dish with a diameter of 100 mm and drying of the suspension was performed at room conditions (22 ± 1 °C and 50 ± 5% RH) to obtain the solid film. The volume needed in the casting process to cover the whole surface of the Petri dish is around 10 mL. The solid CNC films for suspensions of CNC_M, CNC_H, CNC_U, and CNC_H+U were generated using the same method from the corresponding suspensions which have been settled in the room conditions for overnight after each of the specific treatments. The film moisture content was measured after drying the suspension at the room conditions to a constant weight. 

### 2.5. CNC Film Characterization by Ultraviolet-Visible (UV-Vis) Spectrosocpy

The UV-Vis molecular absorption spectra of all the CNC film samples were collected between 190–800 nm at 2 nm spectral resolution using a Genesys 150 UV-Vis Spectrophotemeter from Thermo Fisher Scientific (Waltham, MA, USA). 

### 2.6. CNC Film Characterization by Fourier Transform near Infrared (FT-NIR) Spectroscopy

The FT-NIR spectroscopy was employed to investigate the CNC films and the diffuse reflection spectra between 10,000 and 4000 cm^−1^ were collected using a PerkinElmer Spectrum 400 FT-NIR spectrometer (Waltham, MA, USA) at a 2 nm spectral resolution and 32 scans per sample. Prior to scanning, a reference check was performed on the system using a Spectralon reference standard. The CNC film specimens were then placed on the NIR window with a 0.8 cm diameter which was cleaned for each run using ethanol. The Spectralon reference material was put on top the CNC film specimen during the NIR spectrum acquirement.

Analysis of the FT-NIR spectra to investigate the degree of crystallinity of the CNC films was based on a method developed in the references in the FT-NIR spectra range of 7200–6000 cm^−1^ [43,44]. An assignment of the first overtone of O-H stretching vibration peaks was performed according to the second derivative of the NIR spectra with wavenumber in the range of 7200–6000 cm^−1^ [43,44]. Four regions are identified: amorphous (*A_m_*) region at 7014 cm^−1^, semicrystalline (*S_c_*) region at 6750 cm^−1^, and intramolecular hydrogen bonded crystalline regions (region *C_I_* and region *C_II_*) at 6474 and 6290 cm^−1^. The method developed to evaluate the crystallinity of cellulose structure used the NIR absorption peaks of *A_m_*, *C_I_* and *C_II_* [44] and the relative degree of crystallinity (*CR_NIR_*) was calculated using the equation below:

(1) $$CR_{NIR}=\frac{A\left(C_{I}\right)+A\left(C_{II}\right)\;}{A\left(A_{m}\right)+A\left(C_{I}\right)+A\left(C_{II}\right)}$$

where A(*A_m_*), A(*C_I_*), and A(*C_II_*) are the area under the NIR spectra curve in the range of 7104–7200, 6454–6494, and 6270–6310 cm^−1^.

## 3. Results and Discussion

**Figure 1:**
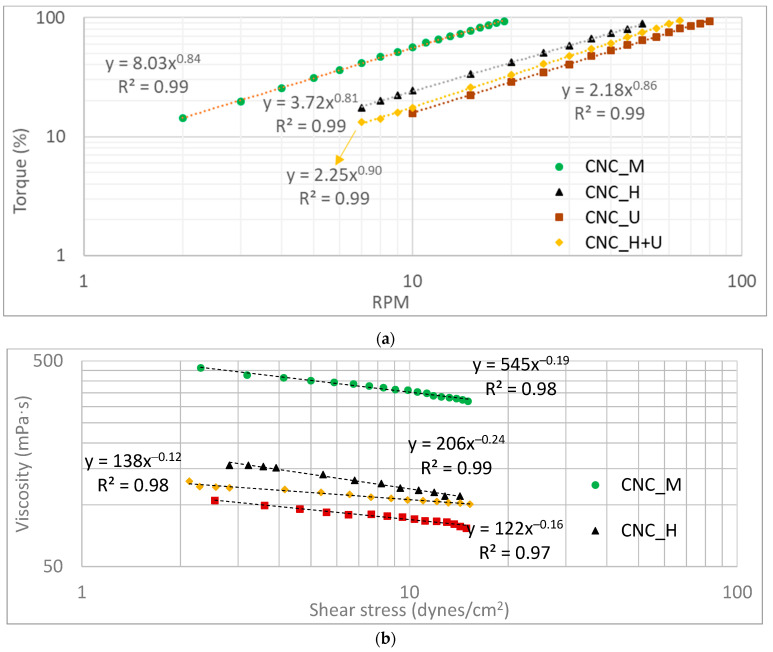

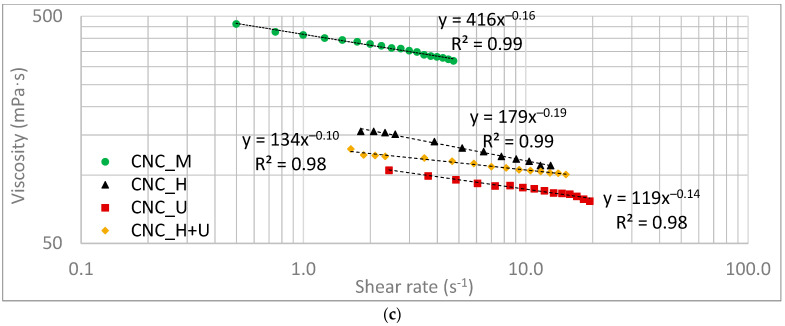
Viscosity measurement results of the CNC suspensions treated by different methods (**a**) torque versus RPM, (**b**) viscosity versus shear stress, and (**c**) viscosity versus shear rate.

The viscosity of the original CNC suspension (CNC_O) was also measured and the primary readings of spindle torque kept changing during the measurement. No consistent readings can be obtained and it is difficult to perform an accurate viscosity measurement. The results are not shown here. The possible explanation is that the random shaking of the suspension during the shipping process impacted the dispersion/distribution/agglomeration of the CNC particles in the liquid which in turn affected the viscosity measurement process. The uneven distribution of CNC particles in the suspension can be visually observed when pouring the CNC suspension into a container. Some locations in the suspension was observed to be more transparent than other locations, demonstrating the different concentrations of the CNC particles in different locations of the container. The viscosity measurement of the other four CNC suspensions treated by different methods generated consistent results and the primary readings from the viscometer for the measurement, including the spindle torque and rotation speed (RPM), are shown in Figure 1a. A power law relationship with a high coefficient of determination (R^2^) between the spindle torque and the spindle rotation speed (RPM) was observed (Figure 1a). Rotating the spindle at the same RPM in different CNC suspensions required different torques. Based on the power law relationships shown in Figure 1a the CNC suspension after mixing by the magnetic stirrer required the highest torque to rotate the spindle when compared with other suspensions. Homogenization and ultrasonication treatments significantly decreased the torques required to rotate the spindle in the CNC suspensions at the same spindle rotation speed.

The viscosity can be directly read from the rotational viscometer. However, the viscosity data from the viscometer is calculated using the primary readings of spindle rotation torque and speed assuming that the liquid is a Newtonian liquid. Obviously, the CNC suspensions are not Newtonian since the relationship between spindle rotational torque and RPM is not linear (Figure 1a). In addition, many research references also showed that the CNC suspensions are non-Newtonian [33,34,35]. Therefore, the viscosity data of the CNC suspensions in this study were calculated based on a template method [45,46,47].

The CNC suspension viscosities (mPa·s) were plotted against shear stress (dynes/cm^2^) and shear rate (sec^−1^) and the relationships are shown in Figure 1b,c, respectively. During the viscosity measurement, similar magnitudes of shear stress were applied (10–90% of the full capacity of the viscometer torque) to all the CNC suspensions. In this specific shear stress range, the viscosities of CNC suspensions decreased with increasing shear stress in a power law model. Under the same shear stress, the suspension of CNC_M has the highest viscosity, followed by the suspensions of CNC_H and CNC_H+U and the suspension of CNC_U has the lowest viscosity. When applying a same external shear stress to the CNC suspensions, the flow behaviors of these suspensions would be different even they have the same concentration of 6 wt.% and the same chemical components. Under the same shear stress of 5 dynes/cm^2^, the viscosity of the suspension of CNC_M, CNC_H, CNC_U, and CNC_H+U are around 402, 141, 95, and 115 mPa·s based on the power law model calculation shown in Figure 1b. When compared with the viscosity of the suspension mixed by the magnetic stirrer, homogenization decreased the viscosity of CNC suspension by almost three times and ultrasonication decreased the viscosity by more than four times. The combination treatment of homogenization and ultrasonication, however, did not generate a direct addition effect on decreasing the suspension viscosity. In the measured shear stress range, the suspension viscosity treated by the combination of homogenization and ultrasonication locates between those treated by homogenization and ultrasonication individually. The relationships between the suspension viscosity and the shear stress are different as well for the suspensions treated by different treatment methods (Figure 1b). The viscosity results demonstrated that different treatments on the CNC suspension significantly impacted their flow behaviors under a same shear stress which needs to be considered in different applications, such as using CNC suspension for making films or coatings.

When a same shear rate is applied to the CNC suspension treated by the different methods significant differences in the apparent viscosities were also observed and this is demonstrated in Figure 1c with the plots of viscosity against shear rate. At the shear rate of 4 s^−1^, for example, the apparent viscosities of the CNC suspensions are 334, 138, 98, and 116 mPa·s for CNC_M, CNC_H, CNC_U, and CNC_H+U. The plot of the viscosity against shear rate in Figure 1c also showed that all the CNC suspensions are a shear-thinning liquid with a fitted power law flow model (Oswald De Waele model) in the shear rate range studied here. The relationship between the viscosity and shear rate and the fitted power law models with high coefficients of determination are included in Figure 1c. In a power law model, the viscosity of the liquid is correlated with the shear rate in the Equation (2).

(2) $$\eta =k{\dot{\gamma }}^{n-1}$$

where *k* is the consistency index and *n* is the flow behavior index. For the CNC suspension, homogenization and ultrasonication treatments significantly reduced the viscosity compared with the viscosity of CNC suspension mixed by the magnetic stirrer and this viscosity change can be represented by the consistency indices in the power law flow models. The consistency indices are much lower for CNC_H (179) and CNC_U (119) when compared with that for CNC_M (416) (Figure 1c). The consistency index in the power law flow model is directly correlated with the viscosity of the suspension [48]. In the shear rate range researched in this study the models obtained in Figure 1c can be used to predict the viscosity of the CNC suspensions. The shear mixing action introduced by the magnetic stirrer can uniformly distribute and/or disperse CNC particles, originally unevenly distributed in the original CNC suspension, to some degree and a consistent measurement using a rotational viscometer can be obtained. A more dramatical shear mixing in homogenization treatment could help to disperse and distribute the CNC particles in the suspension uniformly to a higher degree, resulting in less agglomeration of the CNC particles. According to the rheological theory of rigid particles in a Newtonian liquid [48], the rate of work creating shear of a volume of material is related to the suspension viscosity which is determined by the orientation of the rodlike particles in the suspension. When the long axis of the particle is aligned with the flow direction the lowest rate of work needs to be done to shear the material. On the other side, more unit energy will be needed to shear the suspension when the long axis of the rodlike particle aligns in an angle between 0 and 180 degrees with the flow direction and the highest rate of energy is required to rotate the particle when the long axis is perpendicular to the flow direction [48]. For the CNC suspension, homogenization decreased the viscosity significantly, which indicates that the treatment reduced the barrier to align the CNC particles with the flow direction, either by enhanced dispersion or increased alignment of the CNC particles directly in the suspension. Homogenization is a common practice used to improve particle dispersion in a suspension and the decreased viscosity of the CNC suspension studied here demonstrated that the rate of work required to align the dispersed CNC particles is lower than that needed for agglomerated CNC particles. 

Ultrasonication decreased the CNC suspension viscosity even further compared with that treated by homogenization. However, there is no mechanical action to align the CNC particles during the ultrasonication treatment. The well-known function of ultrasonication is to enhance the dispersion [30,33,36] and decrease the particle size by rupture the rodlike particles into shorter ones [31,32,35]. Both actions help to align the CNC particles along the flow direction when the suspension is sheared by the spindle during the viscosity measurement, leading to lower viscosity readings. For the CNC suspension treated by homogenization first followed by ultrasonication the viscosity in the studied shear rate range is between the viscosities of the suspension treated by each individual method. After homogenization, good CNC particles dispersion and alignment can be achieved and the following ultrasonication could improve the dispersion more and shorten the CNC particles. The combined treatment, however, is not as effective as ultrasonication with respect to lowering the suspension viscosity. The CNC particle alignment caused by homogenization might change the suspension structure, offsetting or diverting the effect of ultrasonication.

The flow behavior index of the CNC suspension also changed after homogenization and ultrasonication, indicating the underlined dispersion, alignment, and rupture of the CNC particles in the suspension. Water is a Newtonian liquid (*n* = 1) and adding rodlike CNC particles drives the flow behavior of the suspension away from the Newtonian flow (*n* < 1 or *n* > 1). The negative value of *n* − 1 (*n* < 1) in the power law models of Figure 1c demonstrated the shear thinning behaviors for all the CNC suspensions. A suspension having a smaller value of *n* (smaller value of *n* − 1) indicates that the flow of the suspension is relatively further away from the Newtonian flow than for the suspension having a larger *n* value. The suspension processed by combination treatment of homogenization and ultrasonication has the largest *n* value (0.90) followed by the suspension treated by ultrasonication (0.86) and homogenization (0.81). The suspension mixed by the magnetic stirrer has the flow behavior index of 0.84. During the viscosity measurement process, the rotation of the spindle sheared the suspension at a much lower shear rate than that applied during the mixing by the magnetic stirrer and the spindle rotation would not change the nature of the suspension during the viscosity measurement. The flow behavior index of 0.84 for the magnetic stirrer mixed suspension reflected the suspension flow property after mixing one hour at 870 RPM using the magnetic stirrer. Homogenization at a much higher rotational speed of 15,000 RPM significantly changed the CNC particle dispersion and alignment, changing the flow behavior index to 0.81. For the treatment of ultrasonication, no further mechanical alignment of the CNC particles occurred in the suspension. However, the CNC particle rupture changed the morphologies and dispersion of CNC [33,35,38], possibly facilitating the particle alignment during the viscosity measurement process with the rotation of spindle which in turn makes the suspension flow relatively more like a Newtonian fluid (0.86) compared with suspension treated by homogenization (0.81). When the CNC suspension was treated first by homogenization, the following ultrasonication increased the flow behavior index further to 0.90 The viscosity of the suspension processed by ultrasonication treatment did not decrease at the same rate with shear rate as that of homogenized suspension (Figure 1c). High shear mechanical mixing increases the viscosity decreasing rate with shear rate while ultrasonication decreases the viscosity decreasing rate with shear rate. The other conclusion may be drawn is that the ultrasonication drives the liquid toward a Newtonian flow while mechanical shearing moves the suspension toward non-Newtonian flow. 

**Figure 2:**
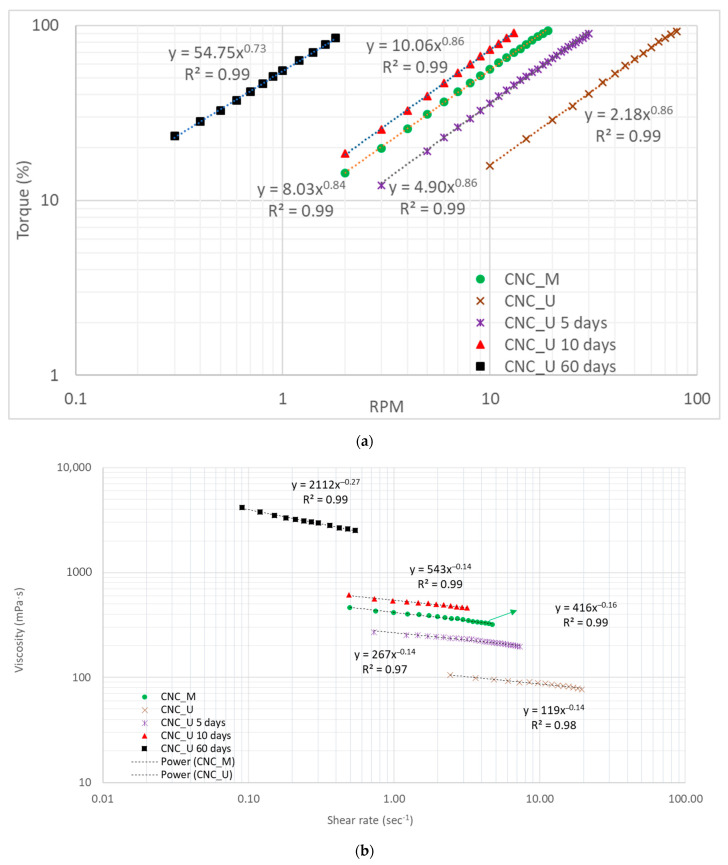
The change of viscosity with time for the CNC_U suspension (**a**) torque versus RPM and (**b**) viscosity versus shear rate.

The apparent viscosity change of the CNC suspension with time after ultrasonication was studied. The CNC suspension settled in a sealed HDPE container was poured into a 600 mL beaker for apparent viscosity measurement and the measurement was conducted at day five, ten, and sixty after the ultrasonication treatment. The primary reading of torque in percentage was plotted against the spindle rotational speed in RPM and the curves are shown in Figure 2a in a logarithm scale. When a same torque was applied to the CNC suspension at different times after ultrasonication treatment, different spindle rotation speeds were obtained. The longer the CNC suspension settled, the lower spindle rotational speed was required to reach the same torque. An exponential relationship was established to represent the torque change with RPM for the CNC suspensions (Figure 2a) and it was observed that the fitted curves in the logarithm scale are parallel with each other up to ten days. There is a slight shifting for the curve at the day sixty and it is not parallel with other curves. 

The viscosity data change of CNC_U suspension with time was calculated and the relationship between viscosity and shear rate is shown in Figure 2b. Power law flow models were observed for all the relationships between viscosity and shear rate measured at different time periods. After sixty days, the viscosity of the suspension increased significantly, and it is more than ten time greater than the viscosity of the CNC_U suspension at day one based on the power law flow model calculation for the same shear rate. The viscosity of the CNC suspension is a dynamic variable, changing with time. After the ultrasonication, the dispersed and ruptured CNC particles could self-assemble into liquid crystalline or colloidal structures [25] and this self-assembly proceeded faster due to the lower viscosity of the suspension after ultrasonication. With the suspension settlement for different time periods various degrees of liquid crystalline or colloidal structures would be formed in the suspension. The strong interactions between the CNC particles in the liquid crystalline or colloidal structures would require much higher energy to rotate or to be sheared along the flow direction, resulting in the increase of the viscosity. The viscosity change can be demonstrated by the consistency indices for the power law flow models. The suspension at different times behaved differently. The flow behavior index did not change for up to ten days (*n* = 0.86) and then decreased to 0.73 at the day sixty. For up to ten days, the viscosity of CNC_U suspension decreases at the same rate with increasing shear rate while at the day sixty, the viscosity decreases at a faster rate with increasing shear rate. The liquid crystalline or colloidal structures formed by self-assembly of CNC particles did not significantly change the shear thinning flow behavior of the suspension for the first ten days and then the higher degree of self-assembly would decrease the flow behavior index gradually until reaching 0.73 at the day sixty.

**Figure 3:**
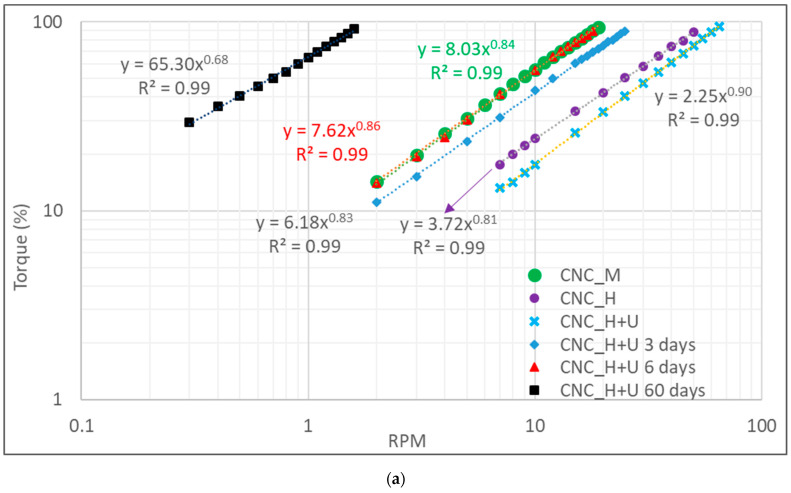

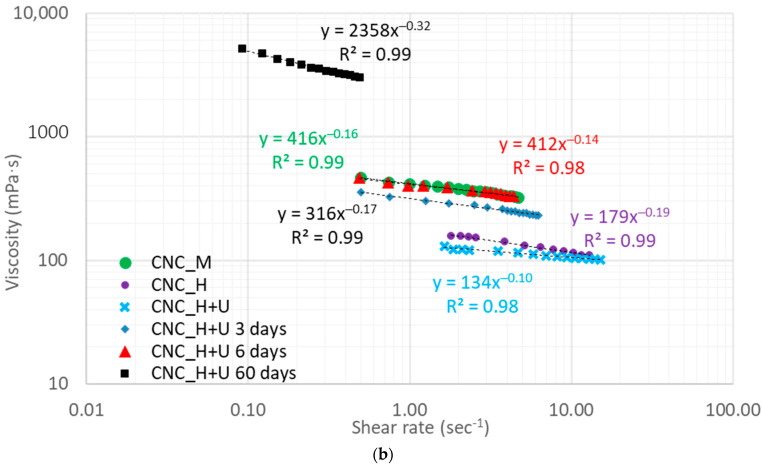
The change of viscosity with time for the CNC_H+U suspension (**a**) torque versus RPM and (**b**) viscosity versus shear rate.

A similar test was performed on the CNC_H+U suspension. The apparent viscosity measurement was conducted on day one, day three, day six, and day sixty. The results for the spindle torque versus RPM are shown in Figure 3a and the plots of apparent viscosity against shear rate are shown in Figure 3b. The results for CNC_M and CNC_H are added for comparison. The CNC_H+U suspension showed the same changing trends with time as demonstrated by the CNC_U suspension. The apparent viscosity of the suspension increased with settling time and at the day sixty, the apparent viscosity is significantly higher than that at day one. The consistency indices in the power law flow models increased from 134, 316, 412, to 2358 from day one, three, six, to sixty. However, the flow behavior indices for the CNC_H+U suspension in the power law flow mode did not keep the same for any time periods and slight change was observed even for day three. The effect of homogenization on suspension viscosity is different than that of ultrasonication and the combined treatment of homogenization and ultrasonication would alter the CNC particle nature existed in the suspension. Therefore, the formation of liquid crystalline or colloidal structures would proceed in a different way than that shown in the suspension treated by ultrasonication. After the treatment, the flow behavior of the CNC suspension kept changing with time and the viscosity of the suspension decreases at different rates with increasing shear rate at different times. Overall, the flow behavior index changed toward a lower number from 0.90 at day one to 0.68 at the day sixty, moving further away from a Newtonian liquid.

**Figure 4:**
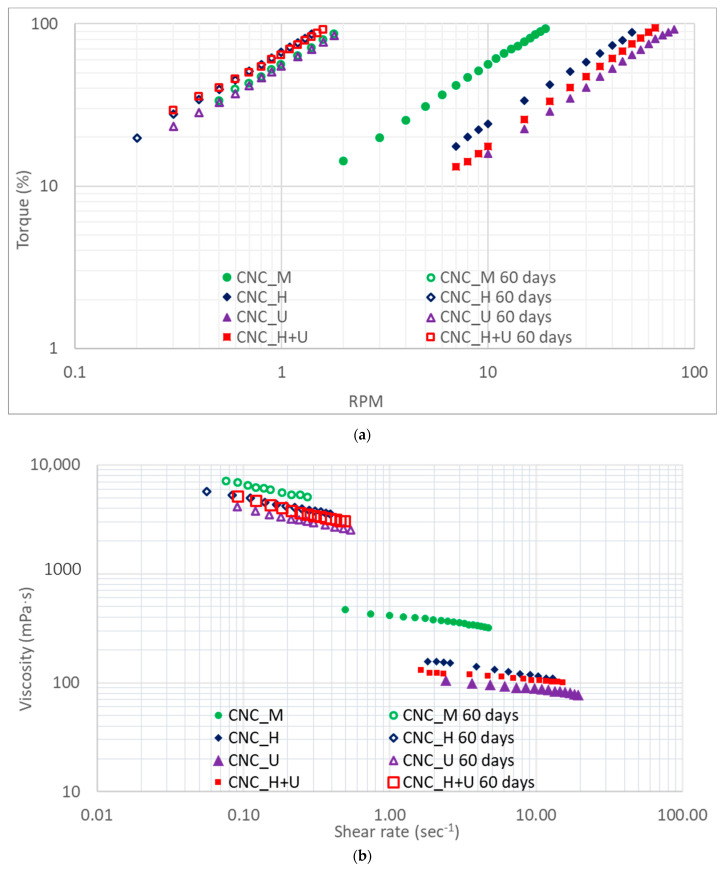
The viscosity of the CNC suspension after settling for sixty days (**a**) torque versus RPM and (**b**) viscosity versus shear rate.

**Figure 5:**
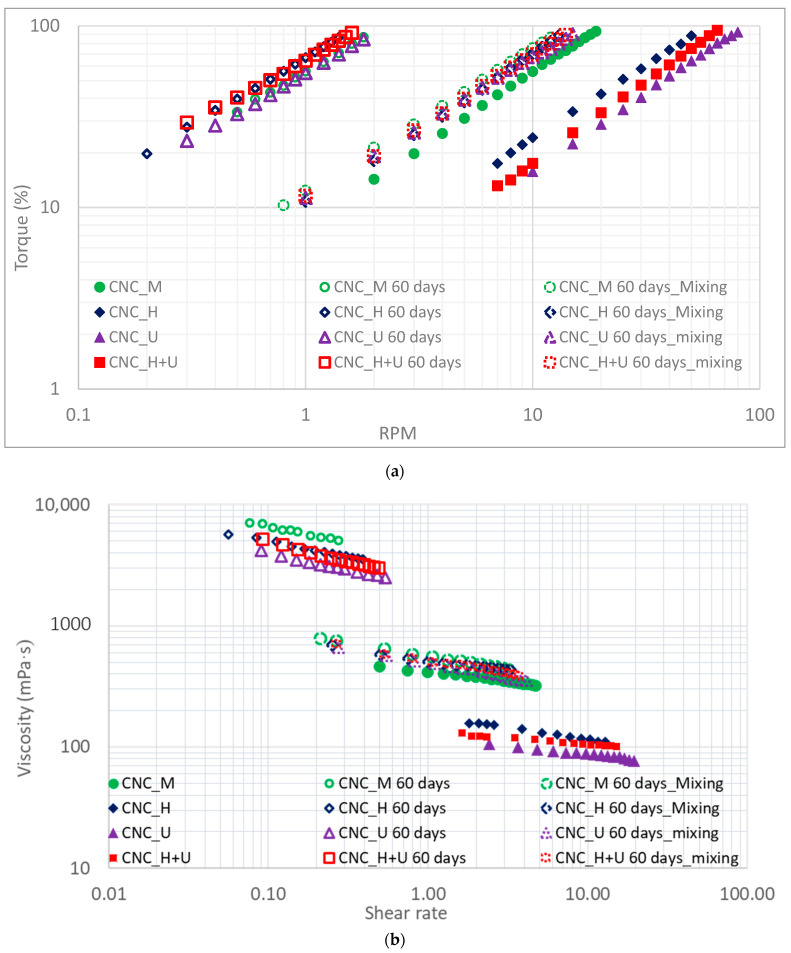
The effect of mixing by magnetic stirrer on the CNC suspension viscosity (**a**) torque versus RPM and (**b**) viscosity versus shear rate.

The CNC suspension stability after different treatments was also evaluated by characterizing the viscosity change with time. After a settlement for sixty days, the viscosities for the suspensions of CNC_M, CNC_H, CNC_U, and CNC_H+U were measured and the primary readings of spindle torque and RPM are shown in Figure 4a. The relationship between viscosity and shear rate are shown in Figure 4b. All the suspensions became significantly more viscous than before. After settlement for sixty days, suspensions of CNC_M, CNC_H, and CNC_U were mixed using a magnetic stirrer for one hour at 870 RPM in closed containers and then sitting at room temperature overnight for the viscosity measurement. The suspension of CNC_H+U was mixed overnight using the magnetic stirrer at 870 RPM for the viscosity measurement. The results for the viscosities are shown in Figure 5. After mixing, the viscosities of all the CNC suspensions were reduced significantly in the tested shear rate range. It can be seen from Figure 5 that one-hour magnetic stirrer mixing of the CNC suspensions decreased the suspension viscosities to the viscosity range near the initial viscosity of the suspension of CNC_M. Overnight mixing of the suspension of CNC_H+U did not lead to a significant difference on viscosity reduction compared with one-hour magnetic stirrer mixing did to the other suspensions. Magnetic stirrer can prepare a CNC suspension with a specific range of viscosity determined by the suspension treatment history.

A quantitative analysis on the viscosity changes for settling and mixing are summarized in Table 1. All the plots of viscosity versus shear rate in Figure 5b for the CNC suspensions were fitted with power law flow models and model parameters are shown in Table 1. Settlement of the suspensions of CNC_M, CNC_H, CNC_U, and CNC_H+U for 60 days increased the consistency indices in the power law models from 416, 179, 119, and 134 to 3568, 2791, 2112, and 2358. The flow behavior indices for all the suspension have also decreased significantly with settlement (Table 1). After mixing with a magnetic stirrer, the consistency indices decreased while the flow behavior indices increased. The consistency indices decreased from 3568, 2791, 2112, and 2358 to 563, 524, 497, and 518 for the suspensions of CNC_M, CNC_H, CNC_U, and CNC_H+U, which are greater than the consistency indices for these suspensions at day one after the treatments. Magnetic stirrer mixing can reduce the suspension viscosity while it cannot reduce the viscosity down to the level that the homogenization and ultrasonication did to the suspensions. This may indicate that the magnetic stirrer cannot disassemble the formed liquid crystalline or colloidal structures as effective as homogenization or ultrasonication. When different treatment methods were applied to the CNC suspension, the CNC material, or the dispersion of the CNC particles in the suspension has been changed, resulting in different flow responses of the liquid when the same shear stress is applied. High shear action of homogenization and high energy sonication are capable of dispersing the CNC particles in suspension with the possibility of dissembling the formed liquid crystalline structures to a higher degree. After initial suspension treatment the consistency indices in the power law flow model of the suspensions are in the order of CNC_M > CNC_H > CNC_H+U > CNC_U and this order kept the same during the settlement and the second time magnetic stirrer mixing treatment (Table 1). This observation possibly indicates that the treatment history of the CNC suspension has an impact on the suspension viscosity.

**Figure 6:**
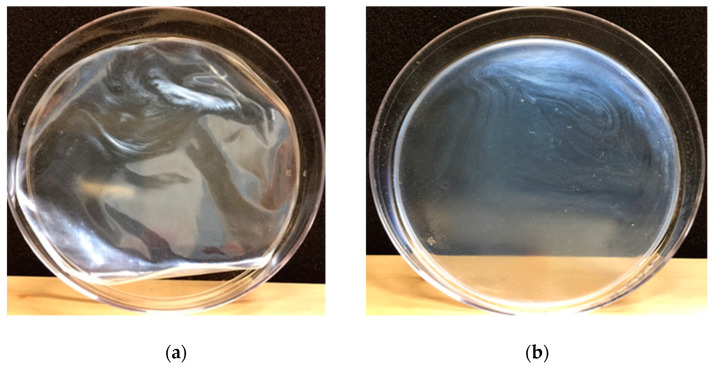
Solidified films from casting solutions (**a**) CNC_O and (**b**) CNC_M.

**Figure 7:**
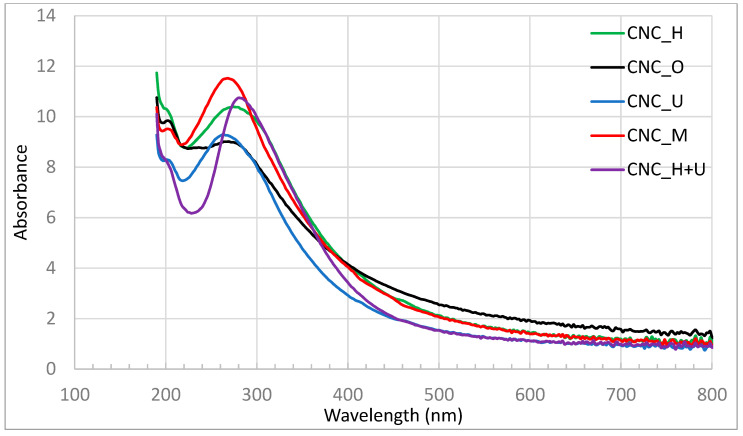
The UV-Vis spectra of CNC films.

Solidified CNC films showed different morphologies when cast from the same suspension treated by different methods. After drying at the room condition to a constant weight, the moisture content of the films was measured to be 8.1%, 7.9%, 8.1%, 8.1% and 8.3% for the films of CNC_O, CNC_M, CNC_H, CNC_U, and CNC_H+U and no significant difference was observed for films generated from suspension treated by different methods. Casting original CNC suspension (CNC_O) formed warping films with deformation wrinkles after drying (Figure 6a). After treatment with different methods, flat films were formed for all the treated suspensions (Figure 6b) and no significant difference among different treatments was observed. The UV-Vis spectra of the solidified films were obtained in the range of 190–800 nm and the spectra normalized by film thickness are shown in Figure 7. Different UV-Vis spectra were obtained for the CNC films after different suspension treatment methods. In the visible light wavelength range from 380 to 740 nm the film of CNC_O showed the strongest absorbance of light while the films of CNC_U and CNC_H+U demonstrated the weakest interference with the visible light. The absorbance spectra in the measurement process are calculated using the transmittance measured by the spectrophotometer and the absorbance signal may include the reflected and scattered light. The film of CNC_O contains many wrinkles and would generate varied reflected and scattered light signals which makes it has the strongest visible light absorption. The absorbance of visible light for the CNC films cast from mechanically treated CNC suspension, including magnetic stirrer mixing and homogenization, is stronger than the films cast from ultrasonication treated suspensions (Figure 7). The CNC_M and CNC_H films have the same visible light absorbance. In the visible light range of 380 to 450 nm, the CNC_U film absorb less visible light than that of CNC_H+U film while in the wavelength range of 450 to 740 nm they showed the same visible light absorbance.

In the UV light range of 190 to 380 nm, significantly different absorbances were observed for these films (Figure 7). Strong absorption below 200 nm is generally observed for cellulose [49] and the same strong absorption can be seen in Figure 7 for these CNC films. Two absorption peaks can be observed in Figure 7 at around 210 and 270 nm for the CNC films of CNC_O, CNC_M, CNC_H, and CNC_U. The relatively weak absorption at 210 nm is associated with the carbohydrate structure of CNC, which can be regarded as being composed of alcohol, ether, and hydrocarbon groups joined by aliphatic bonds [50]. The absorption peak at 210 nm can be identified to be at the similar magnitude in the films of CNC_O, CNC_M, and CNC_H while in the film of CNC_U the intensity of the peak is lower than the other three. The absorption peak at 270 nm in cellulose was explained in different ways from the literature. In acid treated cellulose and regenerated cellulose, an absorption peak at around 270 nm was attributed to the carboxyl groups formed during the treatment [51] while others attributed this absorption peak to the acetal groups in cellulose [52]. CNC is treated by sulfuric acid and oxidation is possible to form carboxyl group. In addition, sulfate ester group was introduced onto CNC surface and sulfate ion demonstrated similar absorption peak at around 270 nm [53]. Therefore, the absorption peak at 270 nm for the CNC films can be ascribed to the carboxyl and sulfate group attached to CNC particles. For the film of CNC_H+U, the UV absorption peak at 210 nm is overlapped with the film of CNC_U and the absorption peak at around 270 nm shifted to around 280 nm after homogenization and ultrasonication treatment of the suspension when compared with other CNC films. The high shear forces induced during the homogenization action followed by ultrasonication could change the structures of CNC differently than each single treatment which would change the absorption spectrum in the UV range, demonstrating the peak shifting. Further investigation is needed to figure out the behind mechanism of the UV absorption peak shifting for the film of CNC_H+U. The peak intensity absorbing at the wavelength of 270 nm can be ranked in the order CNC_M > CNC_H > CNC_U. For the CNC_H+U film, the intensity at the shifted peak is relatively higher than each individual homogenization and ultrasonication treatment. Pre-treatment of CNC suspension using different methods did change the UV and visible light absorption properties. 

**Figure 8:**
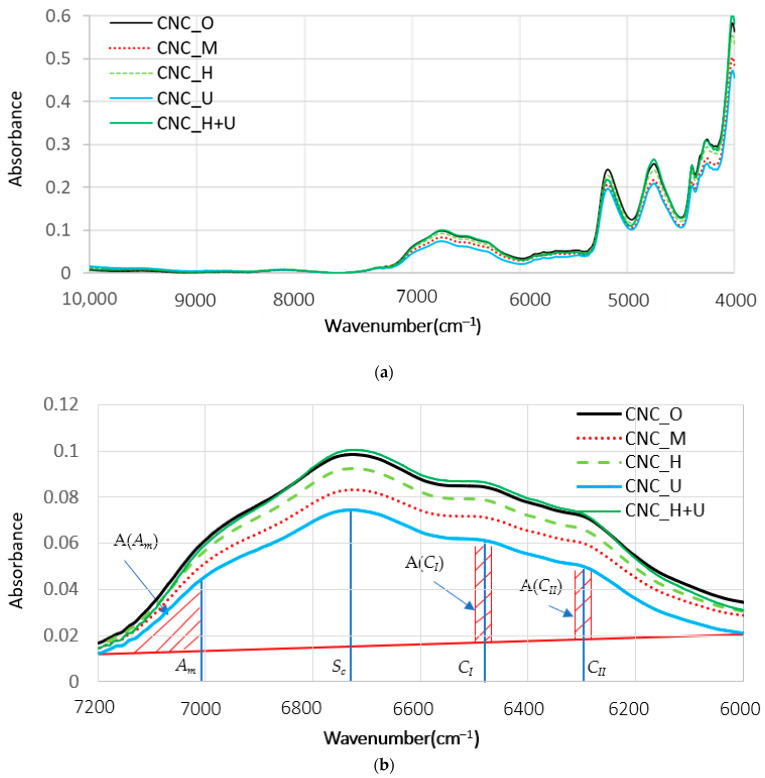
The original NIR spectra of the CNC films (**a**) the whole wavenumber range and (**b**) detail in the range of 7200–6000 cm^−1^.

The original NIR diffuse-reflection spectra of absorbance versus wavenumber for all the CNC films are shown in Figure 8a. In this study, we specifically focused on the absorption bands in the range of 7200–6000 cm^−1^ and the detail spectra in this range is shown in Figure 8b. In this wavenumber range, the spectra are mainly dominated by the first overtone of O-H stretching vibrations [54]. In the cellulose polymer structure, three hydroxyl groups, one primary and two secondary, are attached to each glucose molecule and complex three dimensional structures, including amorphous and crystalline regions, are formed through inter- and intramolecular hydrogen bonds [55]. CNC particles after drying from suspension in air could aggregate into amorphous and crystalline structures due to the formation of hydrogen bonds among available hydroxyl groups. Different suspension treatment methods resulted in different suspension viscosities and are expected to change the hydrogen bonds formation process during drying, resulting in different rates to form amorphous and crystalline structures. The absorption intensity of each peak in the original spectra varied for different CNC films (Figure 8b). Based on the assignments of the four regions for different CNC films, including amorphous (*A_m_*) region at 7014 cm^−1^, semicrystalline (*S_c_*) region at 6750 cm^−1^, and intramolecular hydrogen bonded crystalline regions (*C_I_* and *C_II_*) at 6474 and 6290 cm^−1^, the degree of crystallinity can be calculated using Equation (1). The assigned four peaks and the three shaded areas (A(*A_m_*), A(*C_I_*), and A(*C_II_*)) used for calculating the crystallinity of the CNC films are shown in Figure 8b. A baseline correction was performed using the Spectrum Quant quantitative analysis software and the area under the original NIR spectra was also calculated in the specified wavenumber range using the same software. According to the calculation based on the Equation (1), the degrees of crystallinity for the CNC films of CNC_O, CNC_M, CNC_U, and CNC_H are 57% while the degree of crystallinity of CNC_H+U film is 60%. The CNC suspension treatment through the combination of the homogenization and ultrasonication increased the crystallinity of the dried film and a change in hydrogen bond aggregation was possibly occurred during the film drying process. After the high shear treatment through homogenization better dispersion of CNC particles with a specific alignment could be generated in the suspension. The ultrasonication treatment would then change the dispersion of CNC again and created new surfaces for further aggregation under the preserved CNC particle alignment. Simultaneously, the ultrasonication is performed on the CNC suspension which has a much lower viscosity than suspension treated with magnetic stirrer. The structure change caused by the combination of homogenization and ultrasonication is different than that treated purely by homogenization or ultrasonication. Other treatment showed almost the same crystallinity for the CNC film which may indicate that these treatments did not significantly change the hydrogen bond formation process during the film drying process.

## 4. Conclusions

The rheological behavior of a CNC suspension at 6 wt.% was characterized using a rotational viscometer. In the researched shear rate range, the CNC suspensions treated by magnetic stirrer mixing, homogenization, ultrasonication, and combination of homogenization and ultrasonication demonstrated shear thinning phenomena which were fitted by power law flow models. Homogenization and ultrasonication can significantly decrease the viscosity of the CNC suspension compared with the suspension mixed by a magnetic stirrer. The viscosity of CNC suspension changed with time in a dynamic process after treatment and settlement of treated CNC suspensions in the room conditions increased the viscosity as a function of time. Mixing again with a magnetic stirrer decreased the suspension viscosity significantly to a different level. The flow behaviors of the suspension also changed with treatment and settlement. 

CNC films cast from the suspensions treated by different methods were characterized using UV-Vis spectrophotometer and FT-NIR spectroscopy. Different UV and visible light interferences were observed for the CNC films. Mechanically mixed CNC suspension generated films with higher visible light absorption than films formed from ultrasonication treated suspension, especially in the lower range wavelengths of visible light. The crystallinity characterization using FT-NIR showed that the CNC film obtained from the treatment of homogenization and ultrasonication has the highest degree of crystallinity of 60% while other films have a same degree of crystallinity of 57%. In summary, pre-treatment of the CNC suspension did change the suspension properties and the properties of film formed thereof. The pre-treatment method should be specified for CNC suspension for any downstream application development, including coating, film manufacturing, viscosity modification, etc.

## Figures and Tables

**Table 1:** The power law flow model parameters for the CNC suspensions.

Suspension	Power Law Flow Model Parameter (Consistency Index—*k*; Flow Behavior Index—*n*)					
	Day One		Day 60		Mixing after Day 60	
	*k*	*n*	*k*	*n*	*k*	*n*
**CNC_M**	416	0.84	3568	0.73	563	0.79
**CNC_H**	179	0.81	2791	0.75	524	0.83
**CNC_U**	119	0.86	2112	0.73	497	0.77
**CNC_H+U**	134	0.90	2358	0.68	518	0.79
